# Patented small molecule inhibitors in the ubiquitin proteasome system

**DOI:** 10.1186/1471-2091-8-S1-S14

**Published:** 2007-11-22

**Authors:** Philippe Guédat, Frédéric Colland

**Affiliations:** 1 Hybrigenics SA, 3-5 Impasse Reille, 75014 Paris, France

## Abstract

Deregulation of the ubiquitin proteasome system (UPS) has been implicated in the pathogenesis of many human diseases, including cancer and neurodegenerative disorders. The recent approval of the proteasome inhibitor Velcade^®^ (bortezomib) for the treatment of multiple myeloma and mantle cell lymphoma establishes this system as a valid target for cancer treatment. We review here new patented proteasome inhibitors and patented small molecule inhibitors targeting more specific UPS components, such as E3 ubiquitin ligases and deubiquitylating enzymes.

**Publication history: **Republished from Current BioData's Targeted Proteins database (TPdb; ).

## Introduction

The ubiquitin proteasome system is an important non-lysosomal protein degradation pathway. Ubiquitin is a 76 amino acid polypeptide highly conserved throughout evolution and abundant in eukaryotic cells [1-3]. The conjugation of ubiquitin to protein substrates is a multi-step process involving ubiquitin activation by the E1 conjugating enzyme and its transfer, mediated by ubiquitin conjugases (E2) and E3 ubiquitin ligases, to an internal lysine residue on the substrate [4-7].

**Figure 1:**
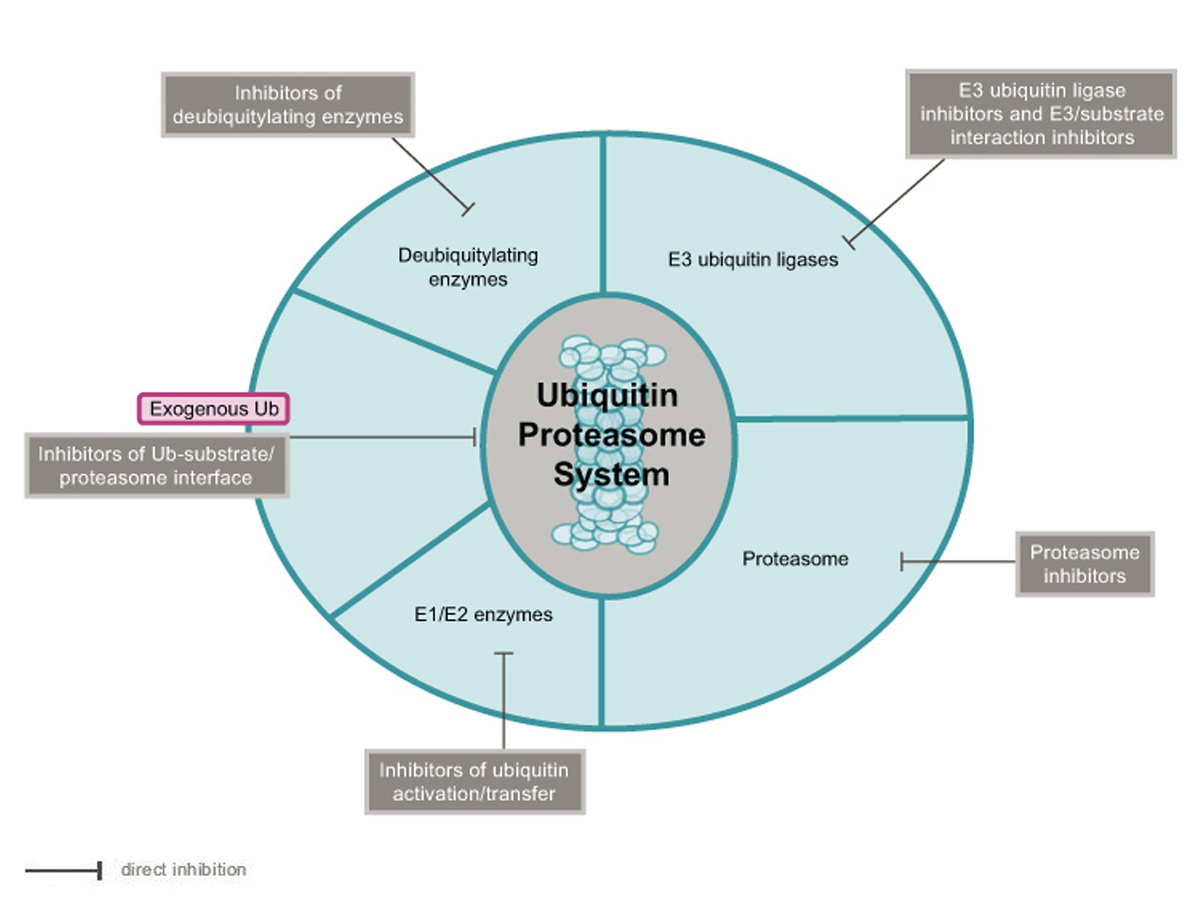
Schematic representation of the ubiquitin proteasome system. The main UPS components are indicated. Patented inhibitors of these components are described in additional data file 1.

The covalent attachment of multi-ubiquitin molecules (linked by lysines at residues 48) to target substrates mostly leads to protein degradation by the multi-catalytic proteasome complex [8-10]. Mono- and polyubiquitylation can be reversed by deubiquitylating enzymes (DUBs), which specifically cleave the isopeptide bond at the C-terminus of ubiquitin [11]. Changes in the function of components of the UPS have been associated with many disease states, including oncogenesis [12], inflammation [13,14], viral infection [15,16], CNS disorders [17,18] and metabolic dysfunction [19]. The involvement of a large number of components in the UPS suggests that there may be many potential target sites for pharmacological interference in the ubiquitin regulatory machinery [20] (Figure 1). In this review, we assess advances in the discovery and development of patented small-molecule inhibitors of the major components of the UPS pathway (see additional date file 1 for recent patent applications related to inhibitors in the UPS), without considering patents on methods or target validation in the UPS.

## Proteasome inhibitors

**Figure 2:**
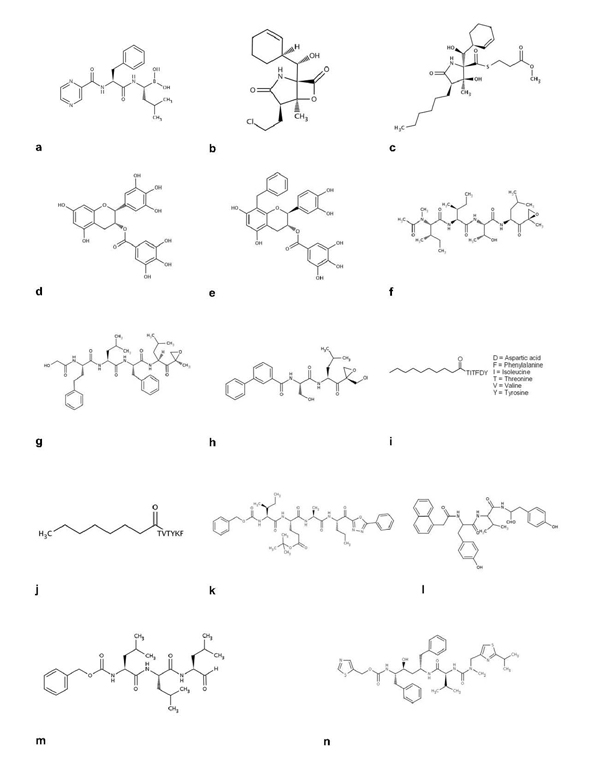
Representative proteasome inhibitors (a-n)

The attachment of ubiquitin to proteins to form K48-linked polyubiquitin conjugates mostly results in proteolytic degradation by a complex cellular structure, the proteasome. Three proteasomal subunits (β 1, β2 and β5) have enzymatic activities, described as chymotryptic-like, tryptic-like and post-glutamyl peptidyl hydrolytic-like [21]. Peptide boronic acids reversibly inhibit the chymotryptic-like activity of the proteasome very efficiently and specifically. One compound, bortezomib (marketed under the name of Velcade^®^[22]), was selected for intensive studies and finally approved by the FDA in 2003 for the treatment of multiple myeloma [10,23] and in 2006 for the treatment of mantle cell lymphoma (Figure 2a). Closely related analogs of bortezomib, such as boronic acid derivatives, benzylmalonic- and amino acid-based derivatives, and boronic ester have been patented as proteasome inhibitors [24,25]. Other compounds with boronic acid or ester function, such as lactam derivatives, have also been patented, with IC_50_ values in the low nanomolar to 100 µM range, without results disclosed [26].

In the wake of bortezomib and its analogs, a second generation of drugs targeting the proteasome is emerging. These drugs include salinosporamide A (NPI-0052) [27,28] (Figure 2b), a secondary metabolite derived from a novel obligate marine actinomycete (*Salinispora tropicana*), and its analogs [29]. NPI-0052 is a highly potent and selective 20S proteasome inhibitor with irreversible activity against all three catalytic sites. In preclinical studies, NPI-0052 has been shown to outperform bortezomib, as it was active against myeloma cells resistant to bortezomib, steroids and thalidomide, and was less toxic to normal cells. Studies using human multiple myeloma xenografts showed that NPI-0052 is well tolerated, prolongs survival and reduces tumor recurrence, thus supporting its use to treat multiple myeloma [30]. A phase I trial was recently initiated in the US, in patients with solid tumors and lymphomas. Open analogs of salinosporamide A, such as 2-pyrrolidone derivatives from microorganisms (*Streptomyces* JS360), have been also described [31]. With a fluorogenic substrate, the most potent compound (Figure 2c) was shown to inhibit human red blood cell 20S proteasome activity *in vitro* with an IC_50_ of 0.2 nM. Other natural compounds, such as (–)-epigallocatechin 3-gallate ((–)-EGCG), the most abundant catechin in green tea, act as chemoprotective and anticancer agents by inhibiting the chymotrypsin-like activity of the purified 20S proteasome *in vitro* (IC_50_ of 0.086 µM; Figure 2d[32]). Analogs of (–)-EGCG have been patented, the most potent of which is a benzilate derivative with an IC_50_ of 0.59 µM against purified 20S proteasome [33,34] (Figure 2e).

A synthetic analog of epoxomicin, PR-171 [35], irreversibly inhibits the chymotryptic site (Figures 2f-2g). Phase I trials are underway, evaluating PR-171 in patients with multiple myeloma and non-Hodgkin's lymphoma. Other analogs of eponemycin and epoxomicin have been also reported to inhibit the proteasome [36]. In this patent, compounds such as ER-805751 (Figure 2h) were classified as bortezomib analogs with the boronic function replaced by an epoxide group. This class of compounds was tested in cell growth, cytotoxicity and proteasomal activity assays, but no specific biological data were presented. However, xenograft studies with ER-805751 were described. This compound inhibits MDA-MB-435 cell growth when administered at doses of 5 and 10 mg/kg, three days per week for four weeks.

Peptides with a sequence of up to 45 glutamine residues linked to aldehyde, boronate or epoxyketone have been shown to inhibit archaebacterial proteasomal activity *in vitro* with minimal effects on mammalian proteasomes, suggesting that they could be used to treat bacterial infections [37]. Lipopeptides and biotinylated-lipopeptides have also been patented as non-covalently bound proteasome inhibitors [38]. The most active of these compounds are lipopeptides (Figures 2i-2j), which inhibit yeast 20S proteasome activity *in vitro* with an IC_50_ of 35 µM. A family of heteroaryl substituted tri- or tetra-peptide derivatives has also been reported to inhibit proteasome activity [39]. The inhibitory efficiency of various compounds, such as that depicted in Figure 2k, was tested on human 26S proteasome activity *in vitro*, human tumor cell proliferation and p21 accumulation in cells. This class of compounds was described as active in these assays but no specific biological data were disclosed. Tyropeptin A analogs have additionally been patented as potent proteasome inhibitors [40]. All the compounds presented in this patent were aldehydes with the potential to act as irreversible analogs. The most potent compound (A7) (Figure 2l) exhibited an IC_50_ of 18.5 nM in HCT-8 human colon carcinoma cells.

Finally, known proteasome inhibitors (bortezomib, MG132 (Figure 2m) and ritonavir (Figure 2n)) have also been patented for additional indications, such as neurodegeneration [41,42], contraception [43], cardiovascular dysfunction [44], fibrosis [45], dry eye disorder [46] and viral infections [47,48], indicating many potential therapeutic indications for proteasome inhibitors. A more exhaustive description of the different classes of proteasome inhibitors, not only restricted to patented inhibitors, has been reviewed in detail elsewhere [10,49].

## E1-conjugating enzyme inhibitors

**Figure 3:**
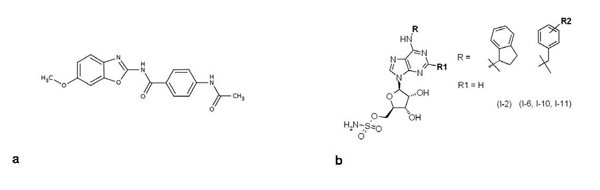
Representative E1 conjugating enzyme inhibitors (a-b)

A family of benzothiazole derivatives has been reported to inhibit the E1 activating agent, preventing the transfer of ATP-activated ubiquitin to different E2 conjugating agents (UbcH10 and UbcH5c) in an *in vitro* fluorescent assay [50]. These inhibitors, which compete for ATP, are illustrated in Figure 3a. Another patent claimed to have identified inhibitors of mouse ubiquitin-activating enzyme E1 (UBE1) by monitoring E2 (Ubc2)–ubiquitin complex formation (IC_50_ below 100 nM) using homogeneous time-resolved fluorescence (HTRF^®^) assays [51] (represented in Figure 3b). Proliferation and *in vivo* anti-tumor efficacy have been assessed for this class of compounds, but the results have not been disclosed. The compounds included in both patents require further investigation, focusing on specificity and cell pharmacology, to confirm that E1 enzymes are relevant targets for the UPS. Indeed, it is likely that multiple pathways could be affected by targeting the E1 machinery and we cannot exclude that many of these pathways could be important for the functions of normal cells.

## E3 ubiquitin ligase inhibitors

E3 ubiquitin ligases form two major classes: (i) the HECT (homologous to E6AP C-terminus) domain E3s, which bind their cognate E2s and transiently accept ubiquitin at a cysteine residue [52], and (ii) the RING (really interesting new gene) domain E3s, which function as scaffold proteins without forming a thiolester intermediate [53]. The RING domain E3s exist as a single peptide or as multiple-component complexes [54].

**Figure 4:**
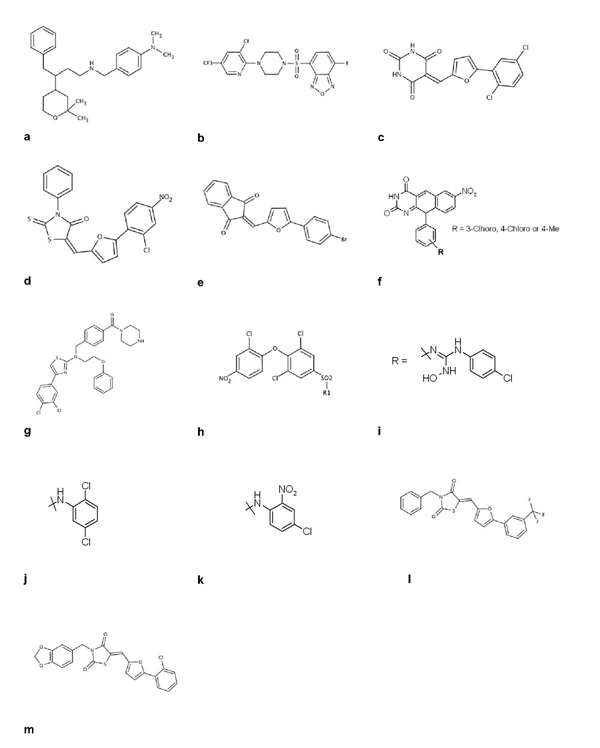
Representative E3 ubiquitin ligase inhibitors (a-m)

Small molecule inhibitors of E3 ligase-mediated p27 ubiquitylation have been patented [55]. However, despite claims that these compounds could regulate ubiquitylation, their *in vitro* activities and the drug target are not presented. Such compounds (compound 17 in additional data file 1; Figure 4a) induce G1 arrest and apoptosis in MDA-MB-435 human breast cancer cells. Several specific E3 ubiquitin ligase inhibitors are discussed below.

### TRAF6 inhibitors

The RING E3 ligase TRAF6 is involved in NF-κB signaling [56]. Since the NF-κB pathway is involved in many important processes such as inflammation and cell survival, inhibition of the ubiquitin ligase activity of TRAF6 could be relevant to the treatment of inflammation and cancers [57]. Several benzoxadiazole derivatives were shown to inhibit TRAF6 autoubiquitylation *in vitro* (IC_50_ below 1µM), in the presence of Uev1A and Ubc13, by ELISA and western blot experiments [58]. These compounds were also shown to affect proliferation of A549 and H1299 cancer cell lines using a cell viability assay (1≤IC_50_<20 µM; Figure 4b).

### POSH inhibitors

POSH is an E3 ubiquitin ligase containing a RING domain. The ubiquitylation activity of human POSH is required to target the HIV Gag protein to the plasma membrane and is essential for HIV type-1 production [59]. *In vitro* POSH autoubiquitylation assays identified 13 series of POSH antagonists [60]. Series 1, 3 and 4 appear the most promising, with IC_50_ values below 1 µM (compounds 19, 20, 21 in additional data file 1; Figures 4c-4e). Interestingly, these compounds are more selective for POSH activity than for the activities of the MDM2, c-CBL and E2 enzymes, and they affect the maturation of HIV particles in a virus-like particle assay (less than 50 % at 3 µM).

### Inhibitors of the MDM2/p53 axis

MDM2, a member of the RING E3 family, is a crucial regulator of p53 stability [61]. MDM2 is amplified or overproduced in many malignant tumors [62], and p53 pathway activation by MDM2 inhibition is well established as a target in the development of new therapeutic agents for cancer [63]. A family of 5-deazaflavin derivatives (including HLI98, Figure 4f) has been identified as inhibiting MDM2 ubiquitin ligase activity [64,65]. These compounds, which stabilize p53 and MDM2, and induce apoptosis, establish proof-of-principle for the inhibition of E3 ligase activity. However, the development of more drug-like analogs is required for future clinical development. Heterocyclic derivative inhibitors of MDM2-mediated p53 ubiquitylation were recently described *in vitro*[66]. These compounds (illustrated here by compound 23 (see additional data file 1) (Figure 4g) and a family including compounds 24a, b and c (see additional data file 1) (Figures 4h-4k)) have been shown to decrease cancer cell proliferation using a cell viability assay and to increase p53-dependent transcription using stable cells carrying a p53-dependent transcriptional regulatory element linked to a luciferase reporter gene. However, the effects of these compounds on autoubiquitylation of APC, a cell cycle-regulated E3 ubiquitin ligase that controls both entry and exit from mitosis, render the interpretation more difficult [67]. Active rhodanine derivatives have also been patented for inhibiting MDM2-mediated p53 ubiquitylation, with an IC_50_ of 74 nM for the most potent compound (Figure 4l) [68]. No cellular data were disclosed, but specific compounds, such as compound 26 (Figure 4m), which inhibits MDM2-mediated p53 ubiquitylation (0.45 µM) more efficiently than APC2/APC11 autoubiquitylation (IC_50_>1000 µM), were identified.

## Inhibitors of the MDM2–p53 interaction

**Figure 5:**
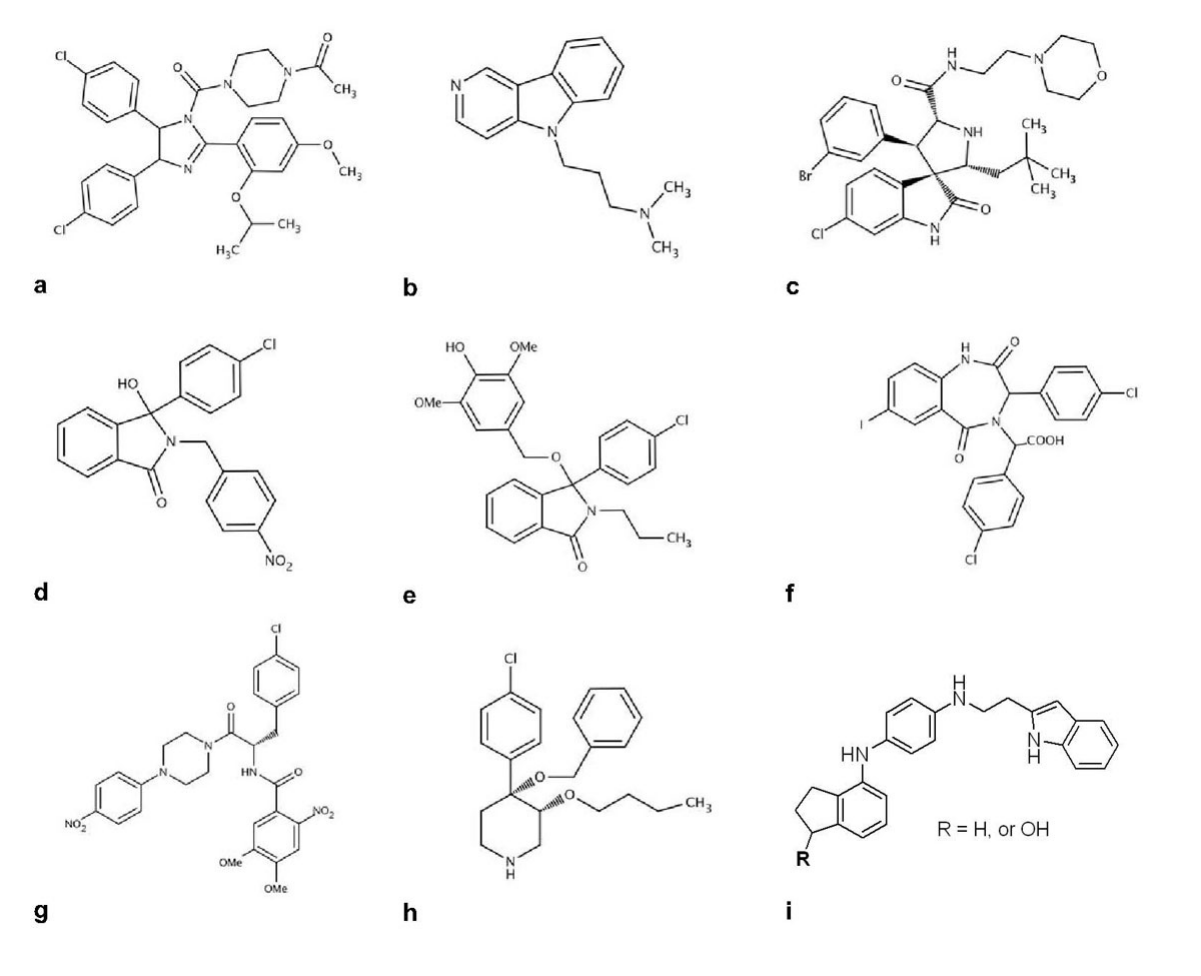
Representative inhibitors of the p53–MDM2 interaction (a-i)

MDM2-mediated p53 ubiquitylation can also be inhibited by directly targeting the protein–protein interaction between p53 and MDM2. Nutlins, which are derived from *cis*-imidazoline (Figure 5a), and were recently identified by high-throughput screening and structure-based optimization, are the first potent and selective patented small molecule inhibitors of the p53–MDM2 interaction [69,70]. These compounds bind MDM2 and inhibit growth of osteosarcoma and colon carcinoma cell lines in a p53-dependent manner. They have also been shown to suppress the growth of established osteosarcoma xenografts in nude mice. Another compound, RITA, was identified in a cell proliferation assay with isogenic cancer cells differing in p53 status (HCT116 colon cancer cells). This compound has been shown to bind p53 *in vitro* using fluorescence correlation spectroscopy and to prevent p53–MDM2 interaction *in vitro* and *in vivo*, thus inducing the accumulation of p53 in U2OS osteosarcoma cell lines [71]. The same group (at the Karolinska Institute) also identified, by ELISA experiments, tricyclic derivatives [72] as inhibitors of the p53–MDM2 interaction and confirmed their inhibitory effects in HCT116 cells; namely decreased p53 ubiquitylation and increased p53 levels (Figure 5b). This compound was also shown to inhibit the growth of HCT116 and U2OS cells in a p53-dependent manner using cell viability and colony formation assays. Design of compounds based on the structure of the MDM2–p53 binding pocket led to the identification of an entirely new class of MDM2–p53 antagonists based on the spirooxindole core structure. Some of these inhibitors specifically inhibit the growth of a p53-positive prostate cancer cell line [73,74]. One such compound, Ke-43 (Figure 5c), inhibits the p53–MDM2 interaction competitively, with an IC_50_ of 18 nM. Interestingly, a correlation between *in vitro* and cellular activity has been demonstrated. Other compounds, such as isoindolin-1-one-based inhibitors or 1,4-benzodiazepinedine derivatives (Figures 5d-5f), have been shown to inhibit p53–MDM2 interaction *in vitro* (in the micromolar range) and to induce p53-dependent gene transcription in cancer cell lines [75-78].

Small molecules, such as substituted 4-Phenylpiperazine [79], or substituted piperidine [80], were identified as antagonists of the p53–MDM2 interaction in ELISA assays *in vitro*, with activity in the micromolar range; however, no cell pharmacology studies were carried out for these molecules (Figures 5g-5h). Several compounds selected for their ability to induce p53 and to decrease cell proliferation in U87MG glioblastoma cells (IC_50_ approximately 10 nM) have recently been patented [81] (compound 35 in additional data file 1; Figure 5i). However, the mechanism leading to p53 upregulation was not described. These families of compounds differ structurally and are leading candidates for the development of anti-cancer drugs.

## Deubiquitylating enzyme inhibitors

Another way to interfere with the UPS is to target the ubiquitin deconjugation system, by inhibiting deubiquitylating enzymes. The human genome encodes approximately 95 putative DUBs [11], divided into five subclasses, of which the USP (ubiquitin-specific protease) and UCH (ubiquitin C-terminal hydrolase) enzymes are the best characterized [11].

**Figure 6:**
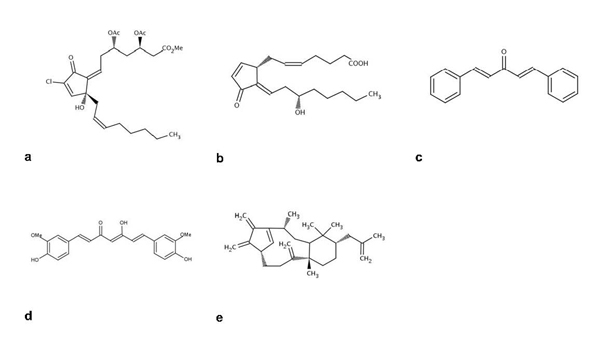
Representative deubiquitylating enzyme inhibitors (a-e)

The first patented small molecule inhibitors of cellular ubiquitin isopeptidases (cyclopentenone PNGs), which have been identified using ubiquitin–PEST and z-LRGG–AMC as substrates, induce cellular accumulation of polyubiquitylated proteins and cause apoptosis in colon cancer cells [82-84] (Figures 6a-6b). Interestingly, cell death correlated with inhibition of isopeptidase activity. However, the existence of any selective inhibition on the various isopeptidase family members has not been disclosed. A molecular determinant conferring activity has been identified in this class of inhibitors, leading to the characterization of additional inhibitors with similar activities, such as dibenzylideneacetone (DBA), curcumin and shikoccin (NSC-302979) (Figures 6c-6e).

USP7 (or HAUSP) silencing results in p53 induction mainly due to the deubiquitylating activity of USP7 on its primary target, MDM2 [85,86]. In addition, USP7 has been recently involved in deubiquitylation of monoubiquitylated FOXO4, thus resulting in FOXO4 inactivation by nuclear export and upregulation of the oncogenic PI3K/PKB signaling pathway [87]. These findings suggest that the targeting of USP7 with small molecule inhibitors could be useful for treating cancer. High-resolution analyses of the structures of USP7 in complex with some of its substrates (MDM2, p53 and EBNA1) have identified a consensus sequence for USP7 binding [88,89]. Recently patented are synthetic inhibitors of USP7 protein binding containing the polypeptide portion P^1^-Gly-P^3^-Ser, where P^1^ is a glutamic acid residue or an amino acid with a non-polar side chain and P^3^ is a glycine residue or an amino acid with a non-polar side chain [90]. High-throughput screening of 65,000 molecules for full-length USP7 cysteine protease activity *in vitro* led to the identification of several drug-like hits [91]. One such molecule, HBX 41,108 (IC_50_ values of 0.53 µM), stabilizes p53 *in vitro*, activates p53 target gene transcription without genotoxic stress in HCT116 colon cancer cells, inhibits cell growth and promotes apoptosis [92].

Ubiquitin-specific protease-8 (USP8) silencing has been shown to induce G1 arrest and apoptosis in several tumor cell lines. Small molecule inhibitors of USP8 (HBX 90,397 and HBX 90,659) have been shown to inhibit HCT116 and PC3 cell growth, and to display specificity for USP8 among a panel of cysteine proteases [91].

Compounds active on USP2 and UCH-L3 have also been identified in a fluorescence polarization assay [93]. The compounds are disclosed to show IC_50_ values ranging from 100 nM to 50 µM for UCH-L3 and 100 nM to 100 µM for USP2. No specific biological data are presented.

## Additional intervention points in the UPS

### Inhibitors of the ubiquitylated substrate/proteasome interface

**Figure 7:**
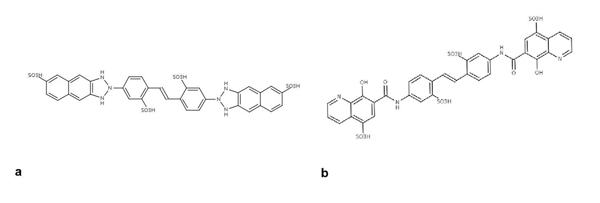
Representative inhibitors of the ubiquitylated substrate/proteasome interface (a-b)

A chemical genetic screen of more than 100,000 compounds in *Xenopus* extracts led to the identification of ubistatins, which block the binding of ubiquitylated substrates to the proteasome [94,95]. Compounds 41 and 42 (see additional data file 1) (Figures 7a-7b) compete with the ubiquitin chain receptors of the proteasome for binding to the ubiquitin–ubiquitin interface of ubiquitylated substrates. Despite their negative charges and lack of cell permeability, ubistatins provide a new therapeutic intervention point in the UPS.

### Exogenous ubiquitin as an immunomodulator

Exogenous ubiquitin has been reported to have anti-inflammatory effects in endotoxic and traumatic shock [96,97], and this discovery has been patented [98]. This immunomodulatory property could be used for new approaches to the treatment of inflammation. Little is known about the mechanism of action of exogenous ubiquitin, but its uptake and conjugation to intracellular proteins have recently been demonstrated, providing a new method of interfering with the UPS after inflammatory stimuli [99].

## Conclusion and new frontiers

The increasing number of patented inhibitors affecting the UPS discovered over the last three years clearly illustrates a growing interest in this system as a target pathway for the development of new therapies. New proteasome inhibitors now in clinical development, such as NPI-0052 or PR-171, appear to be promising candidates to follow on from bortezomib in cancer treatment. The ‘passive’ role traditionally attributed to the proteasome is being challenged by the discovery of proteasome-associated proteins involved in the modulation of proteasome function, such as ubiquitin binding proteins, E3 ubiquitin ligases or deubiquitylating enzymes. In that context, we can hypothesize that targeting these proteins rather than targeting the classical proteasomal catalytic sites may result in distinct phenotypes.

Upstream from the proteasome, the UPS provides many additional targets for small molecule inhibitors (Figure 1). These potential therapeutic intervention points, such as E3 ligases or deubiquitylating enzymes, are now targeted for inhibition by small molecules. The main strategies consist of either selectively blocking the catalytic activity of these enzymes or preventing the enzyme–substrate interaction. Due to the important role of MDM2 in promoting degradation of the p53 tumor suppressor, considerable efforts have been devoted to the identification of MDM2 inhibitors. Some of these compounds, such as Nutlins, appear very promising as cancer therapeutics since they are effective in animal models upon oral administration without noticeable toxicity. These inhibitors of the p53–MDM2 interaction also provide strong support for the therapeutic application of selective disruption of protein–protein interactions. Inhibition of E3 ligase activity is still a significant challenge due to the complexity of the ubiquitin conjugation system. Indeed, even with the less complex (HECT) monomeric E3 ligases (when compared with the multi-components E3s), biochemical assay requires at least two additional enzymes: an E1 and an E2. In this context, counterscreens must be performed to eliminate non-selective hits and thiol-reactive compounds.

Regarding most of the deubiquitylating enzymes, there is no need to supply additional purified proteins for HTS (high-throughput screening) assays. These proteins also appear more “drugable” than E3 ubiquitin ligases since many of them belong to the large cysteine protease family, a family of enzymes presently targeted by more than ten cysteine protease inhibitors at various phases of clinical development/trials for diverse diseases [100,101]. Future studies, particularly aimed at identifying potential substrates of deubiquitylating enzymes, as well as at determining the effects of loss of function of these enzymes, will rapidly advance our understanding of the roles of DUBs. Current efforts on this class of enzymes are also focusing on development of biochemical HTS assays based on cleavage of specific ubiquitylated substrates.

Since proteasome activity is essential for the survival of any cell, proteasome inhibition targets an essential function. As such, bortezomib represents a novel generation of cytotoxics. Unfortunately, it suffers, like most of the other chemotherapeutics, from a narrow therapeutic index. Compounds targeting oncogenic E3 ligases or DUBs could show a better specificity, a lower toxicity and possibly a better potency than proteasome inhibitors.

The current focus for UPS-targeted therapies is cancer, but given the dysregulation of the UPS in many other diseases such as inflammation, metabolic dysfunction, CNS disorders and infectious diseases, it is not unreasonable to expect more drug discovery efforts related to these pathologies in the near future.

## Abbreviations

UPS (Ubiquitin Proteasome System), DUB (Deubiquitylating Enzyme), FDA (Food and Drug Administration), IC_50_ (Inhibitor concentration needed to inhibit 50% of activity), (-)-EGCG ((-)-Epigallocatechin 3-gallate), ATP (Adenosine Triphosphate), HTRF^®^ (Homogeneous Time-Resolved Fluorescence), HECT (Homologous to E6AP C-terminus), RING (Really Interesting New Gene), HIV (Human Immunodeficiency Virus), APC (Anaphase Promoting Complex), AMC (Amino-4-Methyl Coumarin), DBA (Dibenzylideneacetone), USP (Ubiquitin-Specific Protease), UCH (Ubiquitin C-terminal Hydrolase), HTS (High Throughput Screening).

## Competing interests

Philippe Guedat and Frédéric Colland are current employees of Hybrigenics laboratories and authors of Patent No. WO2007017758.

## Publication history

Republished from Current BioData's Targeted Proteins database (TPdb; ).

## Supplementary Material

Additional data file 1 Recent patent applications related to inhibitors in the ubiquitin proteasome system. The data file lists recent patent applications related to inhibitors in the ubiquitin proteasome system, detailing specific target, compound, assignee(s), stage of development and relevant references. Click here for file
